# Re-Examining Mortality Sources and Population Trends in a Declining Seabird: Using Bayesian Methods to Incorporate Existing Information and New Data

**DOI:** 10.1371/journal.pone.0058230

**Published:** 2013-04-03

**Authors:** Tim Reid, Mark Hindell, Jennifer L. Lavers, Chris Wilcox

**Affiliations:** 1 Institute of Marine and Antarctic Studies, University of Tasmania, Sandy Bay, Tasmania, Australia; 2 Wealth from Oceans National Flagship, Commonwealth Scientific and Industrial Research Organisation Marine and Atmospheric Research, Hobart, Tasmania, Australia; Institut Pluridisciplinaire Hubert Curien, France

## Abstract

The population of flesh-footed shearwaters (*Puffinus carneipes*) breeding on Lord Howe Island was shown to be declining from the 1970's to the early 2000's. This was attributed to destruction of breeding habitat and fisheries mortality in the Australian Eastern Tuna and Billfish Fishery. Recent evidence suggests these impacts have ceased; presumably leading to population recovery. We used Bayesian statistical methods to combine data from the literature with more recent, but incomplete, field data to estimate population parameters and trends. This approach easily accounts for sources of variation and uncertainty while formally incorporating data and variation from different sources into the estimate. There is a 70% probability that the flesh-footed shearwater population on Lord Howe continued to decline during 2003–2009, and a number of possible reasons for this are suggested. During the breeding season, road-based mortality of adults on Lord Howe Island is likely to result in reduced adult survival and there is evidence that breeding success is negatively impacted by marine debris. Interactions with fisheries on flesh-footed shearwater winter grounds should be further investigated.

## Introduction

Globally, marine vertebrates, such as seabirds, turtles, fish and cetaceans face a number of significant threats [1]–[2]. These can occur during their time at sea, (e.g.s directed or incidental take), or while on land (e.g. from introduced predators, or the taking of eggs or young) [3]–[4]. It is essential to understand the relative contributions of the various threats to make effective conservation management decisions for species. Seabird species are declining at a faster rate than any other groups of birds [5]–[6]. Seabirds forage over a wide area and use marine and terrestrial habitats, this potentially exposes them to many threats, including increased adult mortality caused by fisheries, effects of pollutants on fecundity and immunity, predation by introduced species, habitat destruction, and the effects of climate change [5]. Seabirds are relatively long-lived animals that exhibit delayed breeding and low fecundity, thus any additional adult mortality will have considerable demographic consequences. This life-history strategy presents challenges for researchers attempting to estimate population dynamics and manage multiple threats. While most studies focus on individual threats to populations, a number of recent studies have highlighted the need to consider multiple threats concurrently in order to improve our understanding of the overall status of a population, and how best to conserve it [7]–[8]. This can be most readily achieved with studies in locations where some level of accuracy on the full range of threats can be obtained.

Flesh-footed shearwaters (*Puffinus carneipes*) are a seabird breeding on Lord Howe Island on Australia's east coast [9]. This population represents 5–14% of the total Australian population [10] and 8% of the world's population [11]. The Lord Howe Island population declined by 19% between 1978 and 2002 [12]. Flesh-footed shearwaters are one of the most common seabird by-catch (non-target mortality) species in long-line fisheries around Australia [13]–[14]. During 1998–2002, an estimated 8,972–18,490 birds were killed in the Eastern Tuna and Billfish Fishery (ETBF) out of an estimated 47,000 individuals [14], leading to the perception that by-catch was the principal factor driving the decline. In recent years, there has been a reduction in the observed by-catch rates, from 0.38 birds/1000 hooks between 1998 and 2002, to between 0.02–0.07 birds/1000 hooks between 2002 and 2007, a decline of over 82% [14]–[15]. In addition, fishing effort has been declining since 2002 [15]. This change has been attributed to the fishery moving north as the principle target species changed from yellowfin tuna (*Thunnus albacares*) to albacore tuna (*T. alalunga*) [16]. In spite of the decline in fisheries mortality, there remains uncertainty whether the population has stabilized, or if other factors are driving a continuing decline [12], [17]–[19].

A number of other issues may have contributed to the decline in the Flesh-footed Shearwater population. First, the area of the breeding colonies on Lord Howe Island declined by 36% between 1978–2002 due to land conversion for agricultural and residential purposes [12]. Second, several roads pass through or adjacent to flesh-footed shearwater breeding colonies, and dead adults and fledglings are frequently found along the roadsides [17].

**Figure 1:**
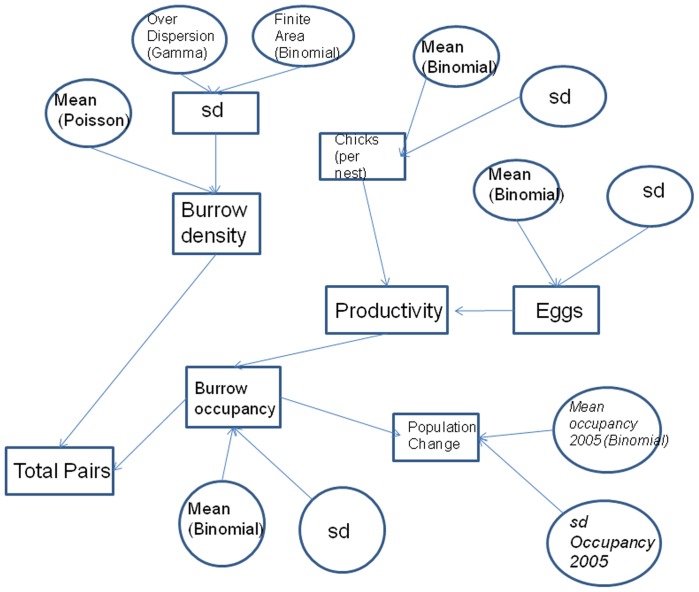
Directed Acyclic Graph. Directed Acyclic Graph of model for estimating flesh-footed shearwater population on Lord Howe Island. Rectangles represent derived variables, while ovals represent parameters that are estimated from data with uncertainty. Variables in italics were taken from the literature (see text). Other variables with parameters took vague priors.

Because only two island-wide censuses of the Lord Howe Island flesh-footed shearwater population have been undertaken (in 1978 and 2002), it is impossible to know if the observed declining trend between those two observations is on-going and hence whether management action is required [12], [19]. Tuck and Wilcox [19] recently developed an Integrated Population Assessment Model to evaluate the status of flesh-footed shearwaters on Lord Howe Island, which suggested the local long-line fishery and habitat issues were currently having little effect on the population. However one of the main limitations with the model included a lack of data on a number of mortality sources (only two, by-catch and habitat destruction were considered). We therefore aim to estimate the effects of some of the other potential mortality sources and make a further estimate of the population of flesh-footed shearwaters on Lord Howe Island so that recent trends in the population can be updated. The analysis is complicated by two factors. First, historical survey data were only available in summarized form as mean population and uncertainty for each colony. Second, recent field data were incomplete, such that not all data was available from all colonies at contemporaneous times. We addressed these issues by developing a Bayesian hierarchical models which allowed us to draw inference across the available information (Fig. 1). Thus, the overall aim of the study was to conduct a new island-wide census of the breeding population of flesh-footed shearwaters on the island, and from this ascertain whether the population was continuing to decline. Further to this we examined possible local sources of mortality other than those previously identified, especially road mortality.

## Results

### Census

**Figure 2:**
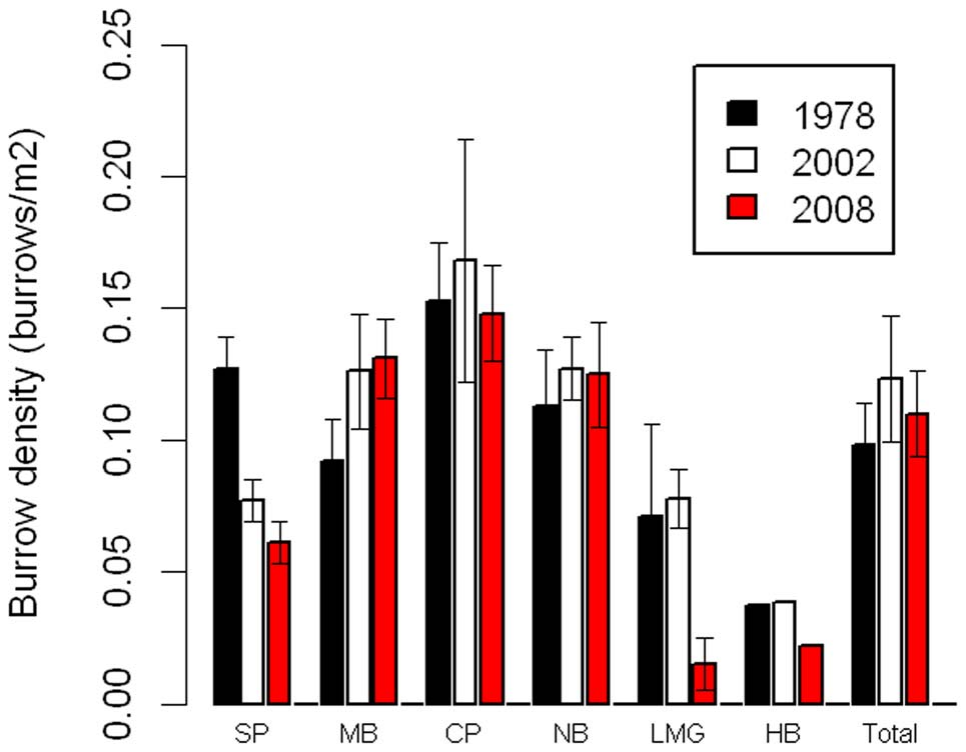
Burrow density changes. Burrow density (burrows m^−2^) at each colony on Lord Howe Island in three years (error bars represent standard errors, as those are what was given in Priddel et al. 2006). (1978 = 1978/9; 2002 = 2002/3; 2008 = 2008/9; SP  =  Steven's Point; MB  =  Middle Beach; CP  =  Clear Place; NB  =  Ned's Beach; LMG  =  Little Muttonbird Ground; HB  =  Hunter Bay).

We estimated the total area of all colonies as 24.73 ha (Table 1), with individual colony size ranging from 0.41 to 7.73 ha. The total area was lower than in 1978/9 (37.75 ha) but similar to 2002/3 (24.31 ha) [14]. Overall, burrow density in 2009 was 0.110±0.008 (posterior standard deviation [PSD, the standard deviation of the posterior estimate]) burrows m^−2^ (Table 1). Burrow density was greatest in the three colonies that were relatively large and enclosed by palm forest (Ned's Beach, Middle Beach and Clear Place), and lowest in the small colonies (Little Muttonbird Ground and Hunter Bay) and at Steven's Point. Burrow density remained generally constant in 2008/9 compared to 2002/3 and 1978/9 (Fig. 2), but with significant declines at Steven's Point and at Little Muttonbird Ground, and possibly Hunter Bay (Fig. 2). Here “significance” is taken to be when the estimates of decline are outside the 95% Credible Interval.

**Table 1:** Area, estimated flesh-footed shearwater burrow density (burrows m^−2^) and number of burrows and 95% Credible Interval for each colony on Lord Howe Island, 2008–2009.

Colony	Area (ha)	Number of transects	Transect area (m^−2^)	No. of burrows	Mean burrow density (burrows m^−2^)	95% Credible Interval	Burrows	95% Credible Interval
Little Muttonbird Ground	0.41	2	560	16	0.015	0.007–0.027	60	29–111
Clear Place	7.73	4	2680	367	0.148	0.115–0.186	11407	8906–14350
Middle Beach	5.88	4	2600	324	0.131	0.103–0.163	7680	6043–9566
Ned's Beach	2.89	1	1240	149	0.125	0.083–0.180	3598	2392–5193
Steven's Point	7.41	5	4000	204	0.061	0.047–0.077	4487	3470–5694
Hunter Bay	0.41						91	
Total							27323	23340–31340

Note all burrows counted at Hunter Bay.

**Figure 3:**
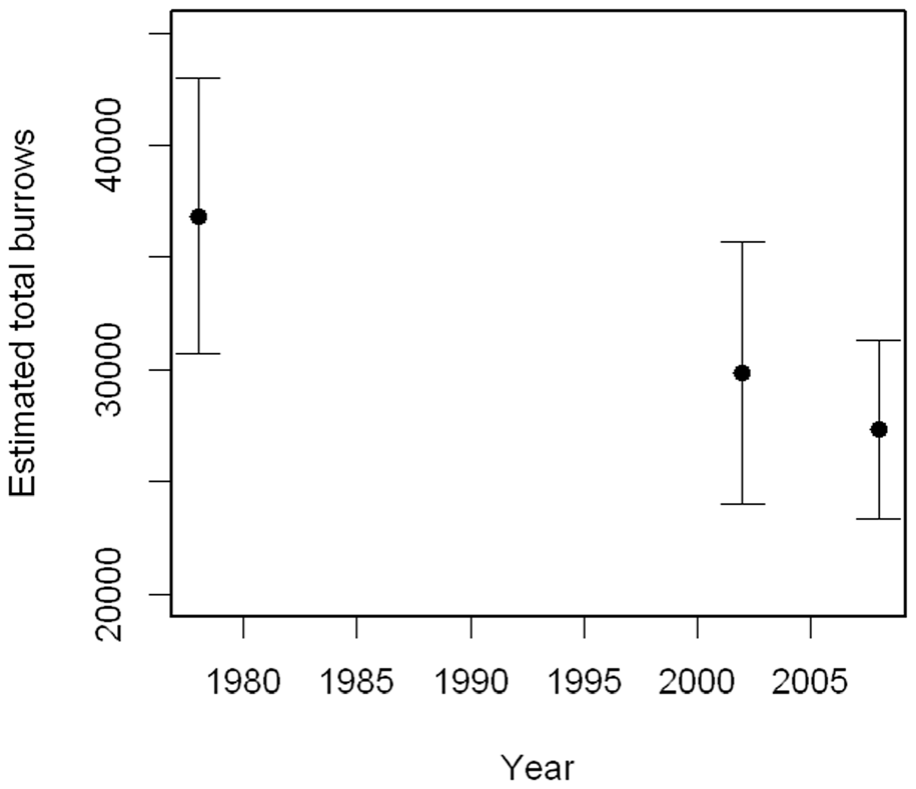
Changes in total burrows. Estimated total number of burrows on Lord Howe Island in 1978/9, 2002/3 [10] and 2008/9.

There were an estimated 27,323 (PSD 2,024) burrows on Lord Howe Island in 2008/9 (Table 1). Most burrows were at Clear Place (42%) and Middle Beach (28%). This represents an 8.5% (95% Credible Intervals −53 to +36%; 65% probability of a decline) decline in burrow numbers since 2002/3, following a decline of 19% (95% Credible Intervals −29 to +55%; 79% probability of a decline) between 1978–2002 (Fig. 3). Most of the decline was due to the estimates for Steven's Point and Clear Place, with marginally greater estimates of the number of burrows for Ned's Beach and Middle Beach compared to 2002/3.

On 27 January 2009, six adult flesh-footed shearwaters were located within a wedge-tailed shearwater colony at Signal Point on the west coast, south of Hunter Bay, where breeding had not previously been recorded. Breeding was not confirmed at this location, but there was some evidence to suggest a small number of nests present (<10) (pers. obs.). No other flesh-footed shearwaters were located in any of the other nearby wedge-tailed shearwater colonies.

### Burrow productivity

**Figure 4:**
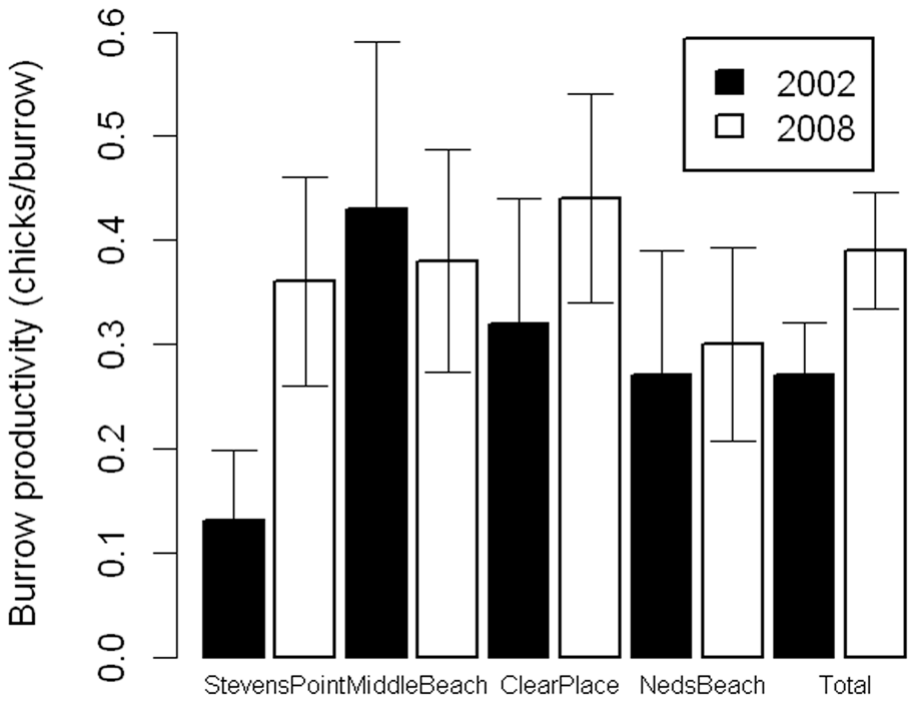
Burrow productivity changes. Burrow productivity (chicks burrow^−1^) at each colony in 2002/3 and 2008/9 (error bars represent standard errors, as those are what was given in [10]).

Burrow productivity was 0.387 (PSD 0.028) chicks burrow^−1^ in 2009 (Fig. 4). Productivity was highest at Clear Place, and lowest at Ned's Beach (Fig. 4). It was significantly higher in 2008/9 than 2002/3. There was no consistent pattern between colonies among years, though it was almost three times higher at Steven's Point in 2008/9 than in 2002/3 (Fig. 4).

### Number of chicks

An estimated 10,571 (PSD 1,114) chicks were produced on Lord Howe Island in 2009 (Table 2). Most chicks were produced in Clear Place (47%) and Middle Beach (27%). This is an 18% increase in the estimated number of chicks produced compared with 2002/3, with most of the increase due to an apparent doubling in the number of chicks produced at Steven's Point [12].

**Table 2:** Estimated number of chicks from each colony on Lord Howe Island, 2008–2009.

Colony	Chicks	95% Credible Interval
Little Muttonbird Ground	30	1–78
Clear Place	4963	3477–6736
Middle Beach	2897	1953–4051
Ned's Beach	1081	627–1711
Steven's Point	1600	1065–2264
Hunter Bay	33	15–51
Total	10571	8495–12850

### Breeding Success

Breeding success (eggs that produced chicks that were likely to fledge) was estimated in Clear Place for three years (2006/7, 2007/8 and 2008/9) (Table 3). Using the Bayesian model to combine these figures with those derived from [12], total breeding success in 2008/9 was estimated at 0.60 (95% Credible Intervals 0.44–0.76).

**Table 3:** Mean (PSD) breeding success (chicks egg^−1^), occupancy (eggs burrow^−1^) and productivity (fledgings burrow^−1^) of burrows in the Clear Place study colony over three seasons.

	Breeding success	Occupancy	Productivity
2006/7	0.76 (0.06)	0.61 (0.07)	0.46 (0.06)
2007/8	0.71 (0.04)	0.70 (0.05)	0.49 (0.05)
2008/9	0.69 (0.03)	0.67 (0.04)	0.46 (0.04)

### Number of Breeding Pairs

Using only the occupancy rate from Clear Place in 2009 to estimate numbers across all colonies, the number of breeding pairs was estimated at 16,794 (2.5–97.5% confidence limits 14,779–18,809) breeding pairs. In contrast, using the Bayesian method with occupancy rates from three years at the Clear Place study site and those provided in [10], the estimated number of pairs is 16,267 (95% Credible Intervals 11,649–21,250). This represents a decline in the number of pairs since 2002/3 of 6.8% (95% Credible Intervals: −21.6–33.3%), with a 70% posterior probability that the number of pairs are declining.

### Estimates of Road-kill

Ten carcasses were found along 40 10 m transects orientated perpendicular to the roads passing through the colonies on 6 April 2009, giving a density of 12.5 (Standard Deviation 0.14) carcasses/1000 m^2^. Most carcasses were located at the end of the transect closest to the road, with six (60%) found within 4 m of the road, and density declining with distance from the road. Twelve transects a significant distance from roads in colonies were surveyed for comparison between 6–10 April 2009 totaling 4,116 m^2^. Only two carcasses were located, giving a density of 0.49 (Standard Deviation 0.37)/1000 m^2^. Thus the density of carcasses adjacent to roads was 25 times greater than that generally found in the colony.

Seven flesh-footed shearwater carcasses that were killed on Ned's Beach Road on the night of 31 December 2007 were marked. Four of these were still easily located and identified on 17 April 2008 (the last occasion when a visit was made), indicating that the carcasses last for at least 3.5 months. Three had disappeared, presumably due to break down or being buried.

Using Equation 4 and assuming a constant rate of mortality and of carcass disappearance we estimate road mortality was 135 birds (95% Credible Intervals 66–233) during the 2008/2009 breeding season. In comparison, using the background density recorded throughout the rest of the colony, multiplied by the colony size, there would be 121 carcasses by natural mortality. This suggests that the road mortality may be more than doubling natural mortality within the colony.

## Discussion

Flesh-footed shearwaters are listed as Vulnerable in New South Wales, as the states only breeding colony (Lord Howe Island) has been declining since 1978 [12]. Fisheries mortality was considered one of the major causes of this decline. It is likely that the majority of the estimated 8–18,000 flesh-footed shearwaters taken in the long-line fishery operating in this area originated on Lord Howe Island due to the proximity of the fishery to the island. Since 2005 there has been a major reduction in the observed mortality of flesh-footed shearwaters on long-line fishing vessels in the ETBF off eastern Australia [14]–[15]. Given this change in mortality, we conducted a census of the population on Lord Howe Island to estimate the most recent population trends.

There is evidence the estimated number of burrows on Lord Howe Island has continued to decline, 19% during 1978–2002 [12] and a further 8.5% between 2002 and 2009, giving an overall decline in burrow numbers of 26% since 1978. Correspondingly, the number of breeding pairs was estimated to have declined 7% between 2002–2008, although the central 95% of the posterior overlapped with zero. Nevertheless, there is a 70% probability that the number of breeding pairs had declined since 2002/2003. If this decline was uniform since 1978 it would equate to 1.3% per annum.

In contrast, the decline in flesh-footed shearwater colony area between 1978–2002 [12] has halted in recent years. Much of this decline was due to land being converted to residential or agricultural uses, and land management approaches on the island have changed in order to reduce impacts on shearwaters [12]. Despite this, the total number of burrows on Lord Howe may have continued to decline during 2002–2008. This was partly driven by the declining density of burrows in recent years, to a density similar to that in 1978. The decline in burrow density was most noticeable in the smaller colonies (Hunter Bay and Little Muttonbird Ground), and the colony most affected by urbanization (Steven's Point). This trend was also noted by [12]. This pattern of declines at the edges of colonies has been noted for other species [20]–[21], and suggests increased conservation efforts may be required there, especially at Steven's Point.

Overall, demographic parameters for the larger colonies (Clear Place, Middle Beach and Ned's Beach) have not changed greatly since 2002, with minimal changes observed at Clear Place and Ned's Beach since 1978 [12]. Middle Beach declined in area by 41% between 1978 and 2002, with an 18% decline in the estimated number of burrows since 1978.

Although the estimated number of burrows and of breeding birds has declined since the previous censuses, the estimated total chicks has apparently increased. These incongruent findings may result from a number of possible, but not mutually exclusive, factors. First, incidental mortality in the longline fishery has declined since the last census in 2002, thus the continuing decline in breeding birds may result from delayed effects of the previous population decline such as the loss of juveniles that would otherwise be recruiting to the population. The increase in chick productivity supports this mechanism, as it may be a result of the reduced food competition among breeders due to a lack of maturing juveniles. Secondly, the contrasting results may reflect the uncertainty inherent in estimating population parameters for a species that nests in deep burrows and are difficult to access. Finally, the results may be due to differences in environmental conditions between years, with 2009 being a particularly good year for chick survival. As this island survey was only carried out for a single year, this is difficult to tell. However, breeding measures for Clear Place were similar in two previous years (Table 3).

We developed a Bayesian model for estimating population size and productivity given partial non-overlapping data sets. We chose this method over a design-based approach to estimating these values for two reasons. First, the modeling approach better accounts for sources of variation [22]. Hierarchical models provide a way to formally incorporate data and variation from different sources into parameter estimates, allowing for the effect of these sources to be combined, but also to be identified [23]. For example, in the current study, estimates of burrow density have errors associated with them that need to be taken into account in estimating numbers of eggs produced. It is therefore important that these errors flow through the model rather than be ignored; hierarchical models are useful for providing a rigorous method for addressing this [24]. In addition, some data were either only partially collected (for example, breeding success in 2008/9 was only collected in Clear Place), or not collected simultaneously, so that data for breeding success existed for all places but not at the same time. Comparison across colonies in years where complete data were available suggested breeding success in Clear Place may be consistently high [12], [25], and so an adjustment to breeding success for 2008/9 was required. This previously collected data could be added either by incorporating it into the likelihood (if the data were available), or by adding the data as a prior using a Bayesian approach (if only the parameter estimates were available as was the case in our study) [24], [26].

### Fisheries interactions and population trends

Fisheries mortality in the Australian longline fishery was a significant source of mortality for flesh-footed shearwaters during 1998–2002 [14]. It has fallen drastically since 2002, therefore on-going declines are unlikely to be related to fishing activities in the ETBF [19]. There is evidence the reduction in fisheries mortality is due to changing fishing areas by the fishery rather than differences in practices, therefore these mortalities may increase again under a different fishing pattern [16]. However, by-catch mortality on the wintering grounds may still pose a significant risk. Flesh-footed shearwaters are known to migrate to the northern Pacific Ocean, with sightings and band records from the Sea of Japan [19], [27], east of Japan [28]–[30], and off western Canada [31]–[32]. In the past, significant numbers of flesh-footed shearwaters have been taken as by-catch in a number of drift net fisheries in the north Pacific Ocean. For instance, in 1987, 116 flesh-footed shearwaters were killed in a salmon drift net fishery east of Japan [33], while in 1990, between 397–957 were killed in neon flying squid drift net fisheries in the North Pacific [30]. While these high seas drift net fisheries closed in the early 1990's, a number of drift (gill) net fisheries continue to operate in national waters, and these continue to pose a potential threat to shearwaters. Japan also operates a large coastal longline fishery in the waters to the east that potentially overlaps with the range of flesh-footed shearwaters which has the potential for significant by-catch, however, no reports on by-catch rates are available [19].

### Road mortality

Flesh-footed shearwater carcasses were much more common along the edge of roads, clearly demonstrating their vulnerability to traffic, with roads more than doubling on-land mortality. Traffic has been highlighted as a problem on Lord Howe Island since the early 1970's [34], and was recently identified as a significant threat to flesh-footed shearwaters [18]. Steven's Point and Hunter's Bay, two of the colonies with declines since 2002, in addition to edge effects, are close to roads. However, one road passes along the edge of Ned's Beach where there is no evidence of decline since 2002. Declines were noted at the small colony of Little Muttonbird Ground, where there are no roads. These patterns suggest while road mortality is important, other issues are also affecting the population.

During 2008/9, 66–233 birds killed on the road represented an annual adult mortality of approximately 0.2–0.7%. This is up to 5 times that recently generated annually through by-catch in the ETBF long-line fishery since 2002, and similar to that estimated for all birds during 2007 [15], [19], [35]. Long-lived seabirds, such as the flesh-footed shearwater, rely heavily on survivorship of breeding adults to maintain population numbers [36]. Using the life expectancy (LE) estimator formula LE  = −1/ln(*φ*), where *φ* =  annual survival, we can estimate the change in mean life expectancy as a result of changes in the survival rate [36]. An adult flesh-footed shearwater with *φ* = 0.92 has a mean life expectancy of 12 years of age while an adult with *φ* = 0.916 (0.2% for roads plus 0.2% for ETBF mortality) would be expected to live only 11.4 years, a reduction of 5% using the minimum estimate of road mortality. The addition of sources of human induced mortality such as those from road mortality and fisheries adds pressure to the survival of seabirds. It is therefore important when these sources are controllable to mitigate their effects. Therefore, small changes in the adult mortality greatly influence the growth of a population by reducing both the lifespan and potential reproductive output of breeding adults.

The flesh-footed shearwater population on Lord Howe Island may have continued to decline during 2002–2008. While habitat destruction and by-catch in the Australian longline fishery have previously been implicated, they are not thought to pose significant ongoing threats to the population. We have demonstrated that there is substantial mortality on the roads of Lord Howe Island at a level similar to that recently observed in the fishery [14]–[15], [19] and the combination of these effects is likely to be important. This suggests management of roads on the island is clearly a concern. There is evidence of plastics in the stomachs of birds, especially chicks, however it is unclear what effect this is having on the population and further investigation is warranted. Finally, flesh-footed shearwaters winter in areas that potentially could interact with fisheries. At present, flesh-footed shearwater by-catch in this region is poorly understood.

## Materials and Methods

Lord Howe Island is a small (1,455 ha) volcanic island located approximately 495 km east of Australia [12]. The island is crescent shaped with a coral reef on the western side. At each end of the island there are volcanic mountains, with the southern ones rising to 875 m. These are separated by an area of lowlands derived from coral-derived calcarinite [12]. Much of this lowland area has been developed for agriculture and settlement. For details of vegetation communities on the island see [37].

**Figure 5:**
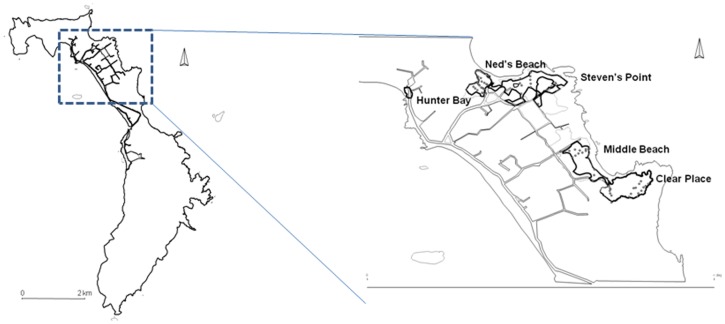
Location of colonies. Map of Lord Howe Island with location of flesh-footed shearwater breeding colonies and roads. Positions of stations used for estimating breeding success marked as circles.

Flesh-footed shearwaters are a medium sized shearwater, weighing between 550–750 g which lay one egg in a burrow [9]. They breed on a number of islands in the southern hemisphere, around New Zealand, in southern Western Australia, on Lord Howe Island, and on Íle Saint-Paul in the Indian Ocean, and migrate to the northern hemisphere for the Austral winter, concentrating in the Arabian Sea [38] and North Pacific [9]. On Lord Howe Island flesh-footed shearwaters breed in lowland areas, predominantly in sandy soil under palm forests on the eastern side of the island. There are currently five discrete colonies on the eastern side (Ned's Beach, Steven's Point, Middle Beach, Clear Place and Little Muttonbird Ground), with a small number also breeding in a single colony on the western side (Hunter Bay) (Fig. 5). Flesh-footed shearwaters are not known to breed on any of Lord Howe's offshore islands [12].

### Census of breeding colonies

A new island-wide census was conducted in 2009, using methods similar to those in the 2002 census by [12]. Fewer data were collected during the census in 1978, with no occupancy or breeding success data recorded [12]. The area of each colony was measured by walking the perimeter with a hand held GPS (Magellan Professional Mobile Mapper 6).

Burrow density was estimated using straight-line transects through each colony on the island (except Hunter Bay where it was possible to count all burrows). Fifteen transects used in this study were previously used by [12]; transects were in total 2.9 km long and covered approximately 6% of colony area. The transects were evenly separated and oriented perpendicular to the longest axis of the colony, passing between colony edges through the centre of the colony. Each transect was divided along its length into 10 meter sections, and all burrows within two meters of either side of each transect were counted. Apparent burrows were scored when they were judged to be large enough for a flesh-footed shearwater to enter (>10 cm long). Based on this survey design, data were divided into 40 m^2^ sections for analysis. The data were treated as count data, with the total number of burrows per section as the response variable for statistical analysis. Fitted values for the number of burrows per transect section were then used to estimate standardized burrow density. Burrow counts were made between 29 October and 5 November 2008, after burrow cleaning had commenced but before egg-laying.

The number of burrows in each colony was estimated by calculating the mean and Posterior Standard Deviation (PSD) density of burrows in each colony from the transect counts, and multiplying that by the colony area (Equation 1) (Fig. 1). The credible intervals were calculated from the posterior.

Here *B,* the total number of burrows, is:
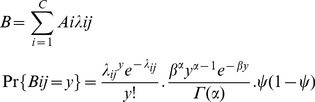
(1)where *A_i_* is the area of each colony *i* and λ*_ij_* is the burrow density of transect *j* of each colony *i*. *B_ij_* is the number of burrows of transect *j* in each of the *i* colonies, drawn from a Negative binomial distribution with parameter *λ_ij_*. Due to limitations in Winbugs we modelled the Negative Binomial as a Poisson distribution with a Gamma overdispersion factor using the density of each colony *i* with *α* and *β* being shape parameters with uniform priors between 0.4 and 2.5. The distribution comes from the Polya distribution [39]–[40].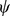 is a probability related to the proportion of the colony surveyed, so that as an increasing number of burrows are counted, the variance of the estimated number approaches zero [23]. A plot of the residuals of the Negative Binomial model against the fitted values indicated an appropriate dispersal.

Burrows within the Clear Place colony have been studied regularly since 2005, with annual counts of the numbers of eggs and fledglings. From these we can derive the breeding success (eggs producing a fledgling) and burrow occupancy (burrows with a pair). Burrow productivity (fledglings per burrow) is estimated as the product of these two quantities. Burrows in this study colony were examined using a custom-made small video camera on the end of a flexible arm [41] in early January each year. As egg laying occurs in early December, it is likely that the January surveys gives a slight underestimate due to egg predation and other losses. Burrows in the study colony were again examined in early April to check for fledgling chicks. Breeding success was estimated from this colony for each year. A small number of burrows were found in April to contain chicks that had not been found to have eggs in January; this was adjusted for using methods outlined by [12].

Burrow productivity was measured in 2009 by checking for the presence of chicks in a sample of burrows in all colonies between 6 April and 10 April 2009. We assumed that because chicks fledge in late April or early May [9], these chicks were likely to survive to fledging and the counts would therefore provide a reasonable estimate of burrow productivity. Three of the transects used in each colony for estimating burrow density were randomly chosen (three new transects were used at Ned's Beach due to considerations of road effects) and six equally spaced stations were created along each transect. The five closest burrows to these stations were examined using the video camera to check for the presence of a chick. Productivity was considered to come from a Binomial distribution (i.e. there are two possible outcomes in a burrow; a chick, or no chick), and this was used to estimate variance and confidence limits.
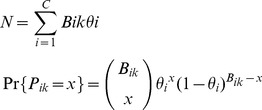
(2)Where *N* is the total number of chicks produced, *B_ik_* is the number of burrows at station *k* in colony *i*, *θ_i_* is the probability of a burrow containing a chick, *P_ik_* is the number of chicks at station *k* in a colony *i* and *x* is the observed number of chicks.

We estimated the number of chicks produced as the product of the number of burrows in each colony, and the productivity of that colony. For Hunter Bay and Little Muttonbird Ground, productivity was not measured and so the mean productivity of the other colonies was used.

We estimate the total number of breeding pairs in each colony as the number of burrows multiplied by the occupancy rate [12], and followed a Bernoulli distribution.
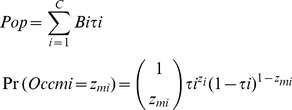
(3)Where *Pop* is the total number of breeding pairs, *B_i_* is the number of burrows in each colony *i*, *Occ_mi_* is the number of pairs in each burrow *m* for each colony *i*, *z_mi_* is the observed occupation of burrow *m* in each colony *i*, and *τ_i_* is the probability of a burrow being occupied in colony *i*. Model dependencies are shown in a Directed Acyclic Graph (Fig. 1). This shows how variables relate to each other and how information and uncertainty flows through the model between levels.

Initially this was calculated using the occupancy rate from the Clear Place study colony. However, this occupancy rate was only derived from one part of one colony, and so may not have been truly representative of the overall occupancy rate and breeding success. A more robust alternative method is to allow previous information to be added to adjust the uncertainty for the derived estimate. We used a Bayesian approach as this provides a framework for combining the occupancy rate for the study colony, and those from other colonies drawn from [12] [23].

The estimated number of breeding pairs is calculated by multiplying the occupancy rate by the number of burrows. In the current study, we had data on the burrow productivity rate for all colonies in 2003 [12] and 2009, and occupancy rates for all colonies in 2003, and for the Clear Place colony in 2007–2009. In order to estimate an overall occupancy rate for 2009 we assumed there was a constant relationship across colonies (with an error distributed as a Student-*t*) between the occupancy and productivity rates, and then used this and incorporated a year effect to estimate the occupancy rate for 2009.

### Estimating road-kill

On 6 April 2009 we measured the density of carcasses along three roads passing through or beside a colony (Ned's Beach Road at Ned's Beach, Skyline Drive and Muttonbird Drive by Steven's Colony) by walking 10 m transects at right angles from the road, counting all carcasses within 1 m of either side of the transect. Ten evenly spaced transects were made along Skyline and Muttonbird Drives, and 20 evenly spaced along Ned's Beach Road (all approximately 10 m apart). Transects were assumed to be representative samples of the full length of the road sides to 10 m. We fit a Poisson GLM to the counts of carcasses on each of the transects, and used this model to predict the density of carcasses at unsurveyed sites, and summing across all locations produced an estimate *M*' of the number of carcasses in the colony at the end of the season. This is an estimate of the number of carcasses beside the road that have not disappeared, and hence is an underestimate of the number killed each year. To estimate the total number of birds killed on the road *M*, we require an estimate of the daily number killed, and the daily number that disappear.

We estimated the disappearance rate of carcasses by marking seven carcasses on 1 January 2008. We re-checked the marked carcasses in early April 2008. Four carcasses remained detectable, and we used this information to estimate the rate of carcass disappearance in order to correct our survey data for carcasses that had disappeared earlier in the breeding season. We have assumed carcasses disappear with daily probability *r* (Equation 4).
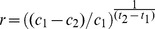
(4)Where *c_1_* and *c_2_* are the number of marked carcasses counted on days *t_1_* and *t_2_*. Life time of corpses was treated as an exponential distribution.

The total number of birds killed during the year on the roads, *M* is the sum of the number of birds killed each day, *M_t_*. However, in order to be counted the carcasses must not disappear before time *t* when the counts were made. Thus the carcasses counted *M*' is the number available to be counted (Equation 5).
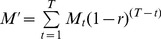
(5)Where *r* is the disappearance rate (from Equation 4) and *T* is the time of the count. *M_t_* can be derived from this equation, as all other variables are known. To estimate a total mortality on the roads, the total area beside the roads to 10 m (the length of the transects) was used.

### Data analysis

We used a hierarchical Bayesian model to estimate the mean and variance of the occupancy and productivity (Fig. 1), rather than estimating each value independently from field data and combining them post hoc, as had previously been used for estimates on Lord Howe Island [12], [42]–[43]. Data analysis was performed using R statistical software [41]. Bayesian analyses were performed using WinBUGS [44] and the R2WinBUGS package in R [45]. The 95% credible intervals for all estimates were derived from the 2.5% and 97.5% quantiles of the MCMC results generated in WinBUGS. Nodes of biological interest were monitored during the running of the model. All runs were made with 100,000 repetitions with a burn in period of 50,000. Five chains were run concurrently with a thinning of 10. Each estimated variable had an Rhat value of 1.00 (where 1 is equivalent to convergence) and effective sample sizes of 15–25,000 (with 25,000 simulations saved).
